# Supplementary material for: The IAP family member BRUCE regulates autophagosome–lysosome fusion
Source: Nat Commun. 2018 Feb 9;9:599. doi: 10.1038/s41467-018-02823-x (PMC5807552; doi:10.1038/s41467-018-02823-x)
Supplement: Supplementary file 1 — Supplementary Information [file 41467_2018_2823_MOESM1_ESM.pdf]

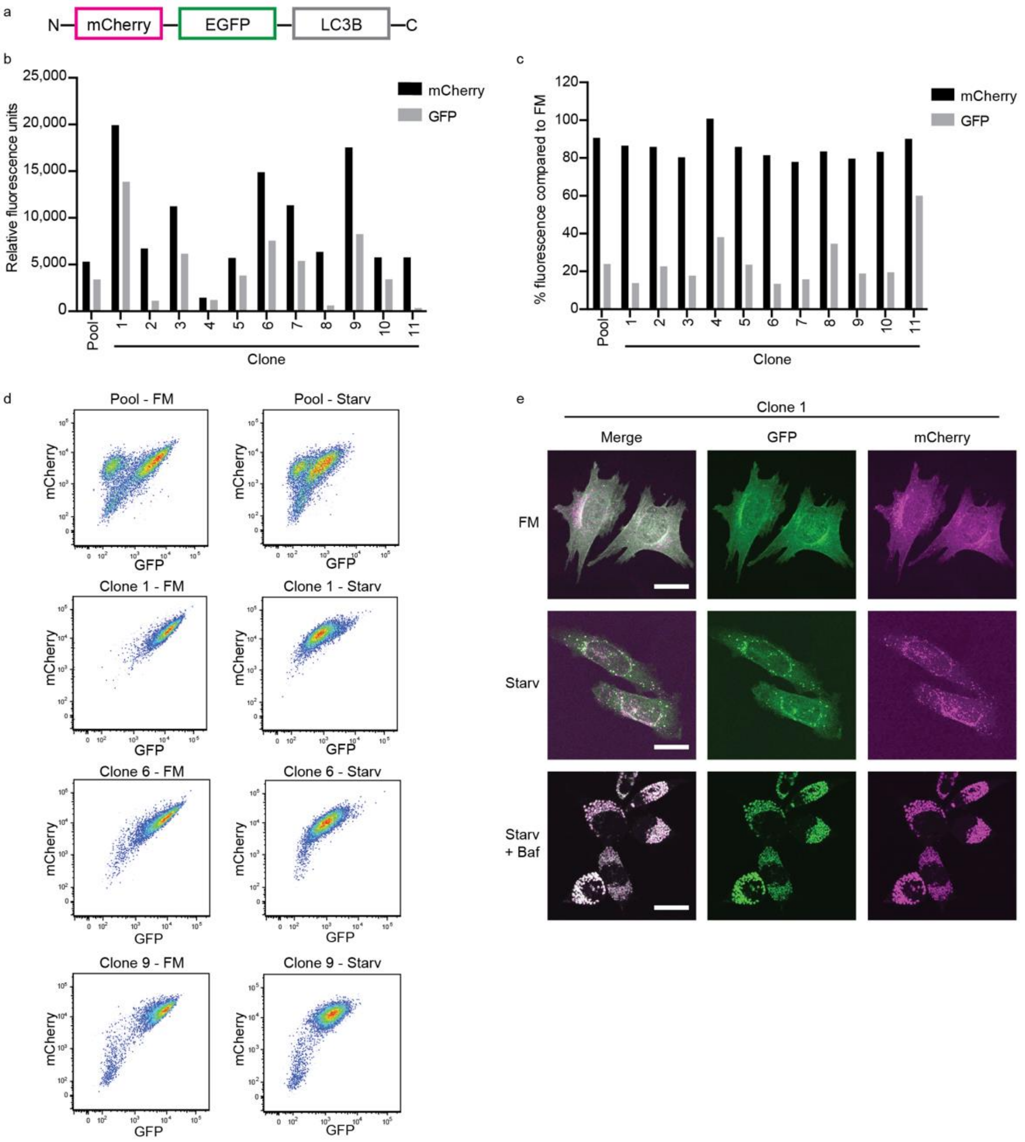

Supplementary Figure 1. Clonal mCherry-EGFP-LC3B expressing MEF line 1 shows loss of GFP signal upon starvation. (a) Schematic representation of a tandem fluorescent mCherry-EGFP-LC3B reporter. **(b)** Relative fluorescence units of mCherry and GFP measured by flow cytometry in 11 single cell-derived monoclonal mouse embryonic fibroblast (MEF) lines (clone 1-11) and a polyclonal line (pool) stably expressing mCherry-EGFP-LC3B, cultured in regular medium. Data are presented as mean from two biological replicates. **(c)** Poly and monoclonal MEF lines were starved for 6 h in Earle's Balanced Salt Solution (EBSS) and the percentage of mCherry/GFP fluorescence units under starvation condition normalized to fully supplemented medium (FM) condition is shown. Data are presented as mean from two biological replicates. **(d)** mCherry/GFP ratios of total cell populations of three monoclonal lines (clone 1, 6 and 9) and a polyclonal (pool) line, grown in fully supplemented medium (FM) or starvation medium (Starv) for 6 h. **(e)** Confocal microscopic images of mCherry-EGFP-LC3B expressing clone 1 in FM, after 6 h starvation, with or without Bafilomycin A1 treatment (Baf; 100 nM). Scale bars, 20 µm.

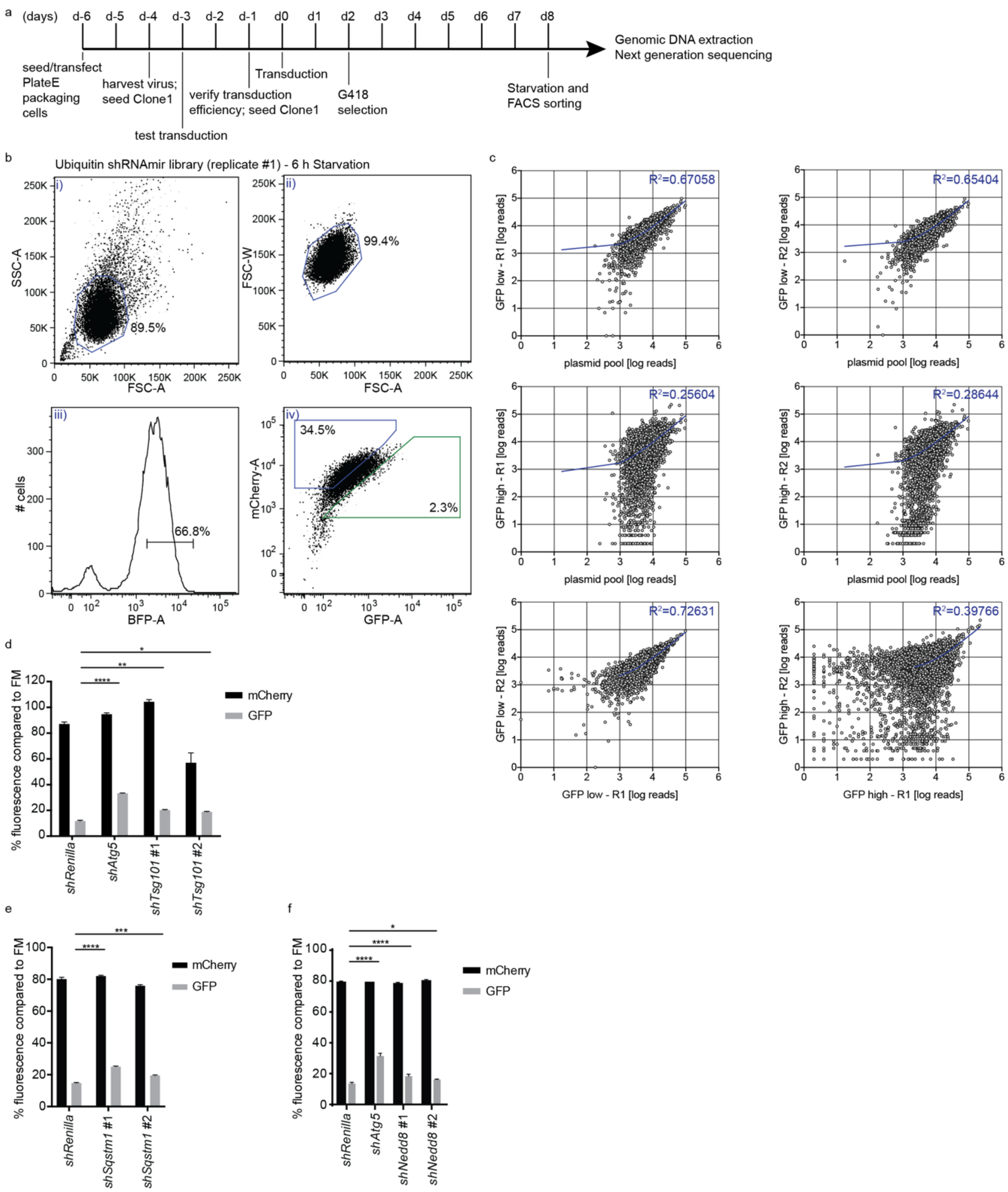

Supplementary Figure 2. Screening of a ubiquitin shRNAmir library based on an mCherry-EGFP-LC3B reporter cell line reliably identified autophagy regulators. (a) Schematic of timeline summarizing the screening process. Retrovirus was generated in Plat-E packaging cells and harvested for test transduction on day -4 (d-4) to estimate the transduction efficiency. Large scale transduction of mCherry-EGFP-LC3B expressing Clone 1 mouse embryonic fibroblasts (MEF) took place on day 0 (d0). Transduced cells were selected by G418 from day 2 (d2), and were starved and sorted by FACS based on the GFP signal on day 8 (d8). Subsequently, genomic DNA was extracted, and analyzed by next generation sequencing. **(b)** Gating history (from i) to iv)) of ubiquitin shRNAmir library transduced cells (replicate #1). After 6 h starvation, cells were trypsinized and sorted based on “GFP low” (blue), and “GFP high” gate (green). Percentage of cells (from parental gate) is indicated. **(c)** Correlation plots of the log10 of normalized reads in different next generation sequencing samples, displayed with trend line and R^2 value. “GFP low” population of both replicates correlates well with original plasmid pool (used for retrovirus

generation), as opposed to "GFP high" population. Replicate #1 and #2 correlate well in "GFP low" population, with lower correlation in "GFP high" population. **(d-f)** Individual knockdown MEF lines were generated (in Clone 1) targeting selected hits with less well understood role in starvation induced autophagy, based on results from Table 1. Remaining % of mCherry and GFP signal in MEFs after 6 h of starvation compared to FM condition is displayed. shRNA identification numbers: *shRenilla*: Renilla.713, *shAtg5*: Atg5.1063, *shTsg101#1*: Tsg101.1332, *shTsg101#2*: Tsg101.336, *shSqstm1#1*: Sqstm1.1895, *shSqstm#2*: Sqstm.1727, *shNedd8#1*: Nedd8.576, *shNedd8#2*: Nedd8.86. Data are presented as mean±SD from three biological replicates (**** $p<0.0001$, *** $p<0.001$, ** $p<0.01$, * $p<0.05$). Representative data are shown from two to four independent experiments.

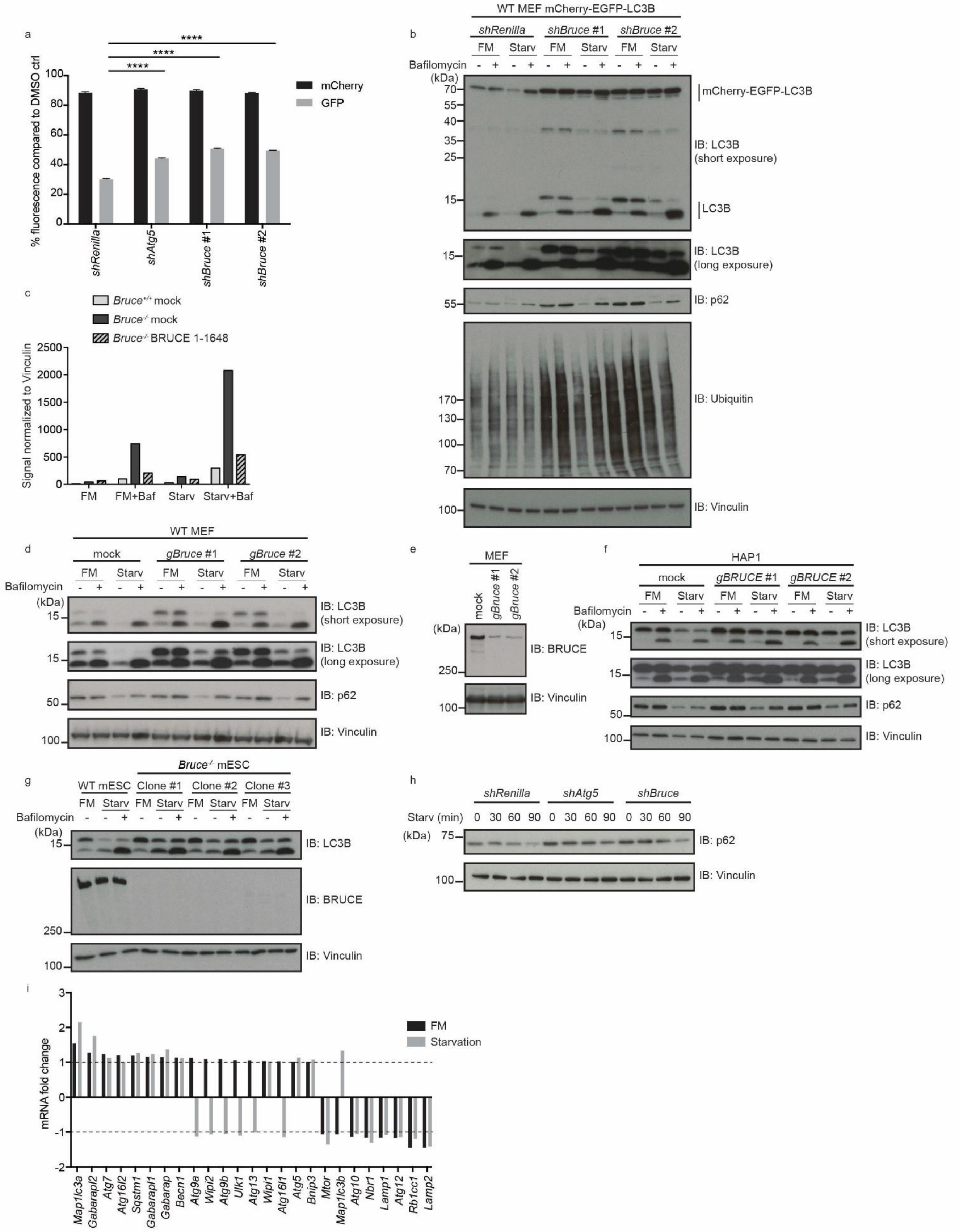

Supplementary Figure 3. BRUCE deficient cells show defects in starvation-induced autophagy and selective mitophagy induction. (a) Normalized mCherry/GFP signals in MEFs stably expressing

mCherry-EGFP-LC3B, treated with Antimycin A/Oligomycin to induce mitophagy, compared to vehicle control (DMSO). KD MEFs, as indicated treated with DMSO or Antimycin A/Oligomycin for 24 h, were examined using flow cytometry. Data are presented as mean±SD from three biological replicates (**** $p<0.0001$). Representative data are shown from three independent experiments. **(b)** Protein levels of exogenous mCherry-EGFP-LC3B, and endogenous LC3B, p62 and ubiquitin in BRUCE knockdown cells. Total cell lysates extracted from *shRenilla*, *shBruce* #1 and #2 clone 1 MEFs in fully supplemented medium (FM), starved for 2 h (Starv) with or without Bafilomycin A1 (100 nM) were analyzed by immunoblotting using antibodies as indicated. **(c)** Quantification of LC3B-II signal in *Bruce*$^{+/+}$ (mock), *Bruce*$^{-/-}$ (mock or BRUCE 1-1648-reconstituted) MEFs normalized to loading control (Vinculin), determined by Odyssey semi-quantitative immunoblotting methods. **(d-f)** Protein levels of endogenous LC3B, p62 and BRUCE in CRISPR/Cas9 mock control and BRUCE mutant MEFs (d) or human haploid cell line HAP1 (f) in FM or starvation condition, with and without Bafilomycin A1 (100 nM). Total cell extracts were analyzed by immunoblotting using antibodies as indicated. BRUCE depletion efficiency in mutant clones was compared to mock treated MEFs. Loading was examined by anti-Vinculin antibody (e). **(g)** Protein levels of endogenous LC3B and BRUCE in parental (WT) and gene trap *Bruce*$^{-/-}$ mouse haploid embryonic stem cells (mESCs). Total cell lysates from WT and *Bruce*$^{-/-}$ (clone #1 - 3) mESCs in FM, starved for 2 h (Starv) with or without Bafilomycin A1 (100 nM) were analyzed by immunoblotting using antibodies as indicated. **(h)** Starvation time course of protein levels of endogenous p62 in *shRenilla*, *shAtg5* and *shBruce* MEFs determined by immunoblotting using antibodies as indicated. **(i)** RNA-Seq data of genes involved in autophagy regulation in *shRenilla* and *shBruce* expressing MEFs in FM and 2 h starvation. Fold change of poly (A)-enriched total RNA in *shBruce* MEFs was compared to *shRenilla* control MEFs.

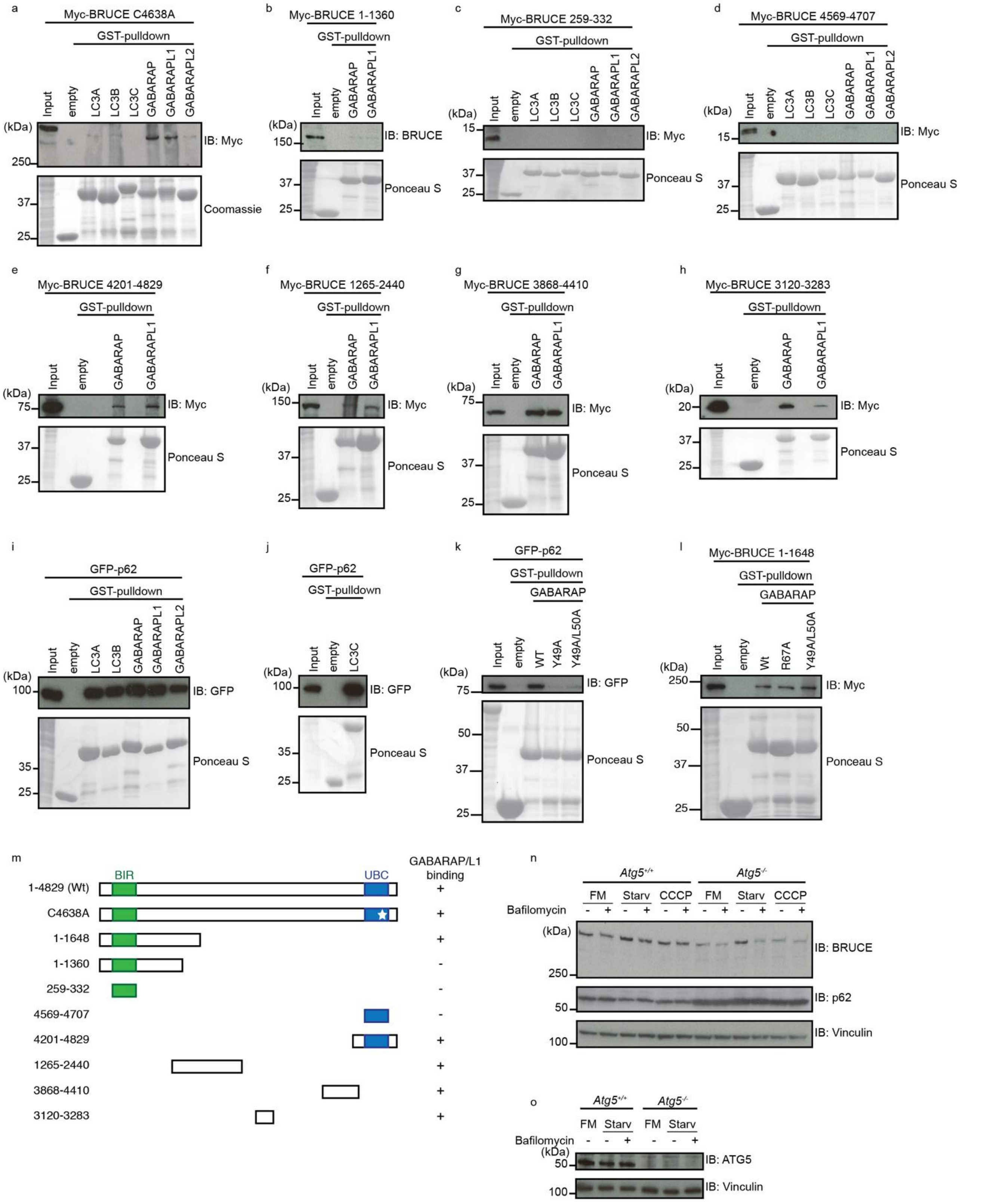

Supplementary Figure 4. Various BRUCE mutants interact with GABARAP and GABARAPL1. (a - h) Interaction of BRUCE mutants with mammalian ATG8 proteins examined by GST-pulldown assays. Total cell lysate of HEK293T cells transfected with Myc-BRUCE mutants as indicated was incubated with GST-ATG8 proteins immobilized with glutathione sepharose beads. Interaction was monitored by immunoblotting using anti-Myc or anti-BRUCE antibody. The amount of GST-proteins used was determined by Ponceau S staining or Coomassie gel staining. **(i and j)** Interaction of p62 with mammalian

LC3 proteins examined by GST-pulldown assays. Total cell lysate of HEK293T cells transiently expressing GFP-p62 was incubated with GST-ATG8 proteins immobilized with glutathione sepharose beads. The amount of GST-protein used was compared by Ponceau S staining. **(k)** Interaction between p62 and GABARAP (WT, Y49A and Y49A/L50A) examined by GST-pulldown assay as in (i-j). **(l)** Interaction between BRUCE (1-1648) and GABARAP (WT, R67A and Y49A/L50A) examined by GST-pulldown assay as in (a-h). **(m)** Schematic representation of BRUCE mutants with indicated positive (+) or negative (-) binding to GABARAP/GABARAPL1. The baculovirus inhibitor of apoptosis protein repeat (BIR) domain is displayed in green, and the ubiquitin conjugating (UBC) domain in blue. C4638A is a point mutant rendering BRUCE catalytically inactive. Deletion mutants are indicated with amino acid numbers. **(n)** Protein levels of BRUCE and p62 in *Atg5*$^{+/+}$ and *Atg5*$^{-/-}$ MEFs. Total cell extract of cells in full medium (FM), starved for 2 h (Starv) or treated with CCCP (20 µM), with or without Bafilomycin A1 (100 nM) were assessed for immunoblotting using antibodies as indicated. Vinculin levels were monitored as a loading control. **(o)** Protein levels of ATG5 in *Atg5*$^{+/+-}$ and *Atg5*$^{-/-}$ MEFs examined by immunoblotting. Cells were grown in FM or starved for 6 h (Starv), with or without Bafilomycin A1 (100 nM).

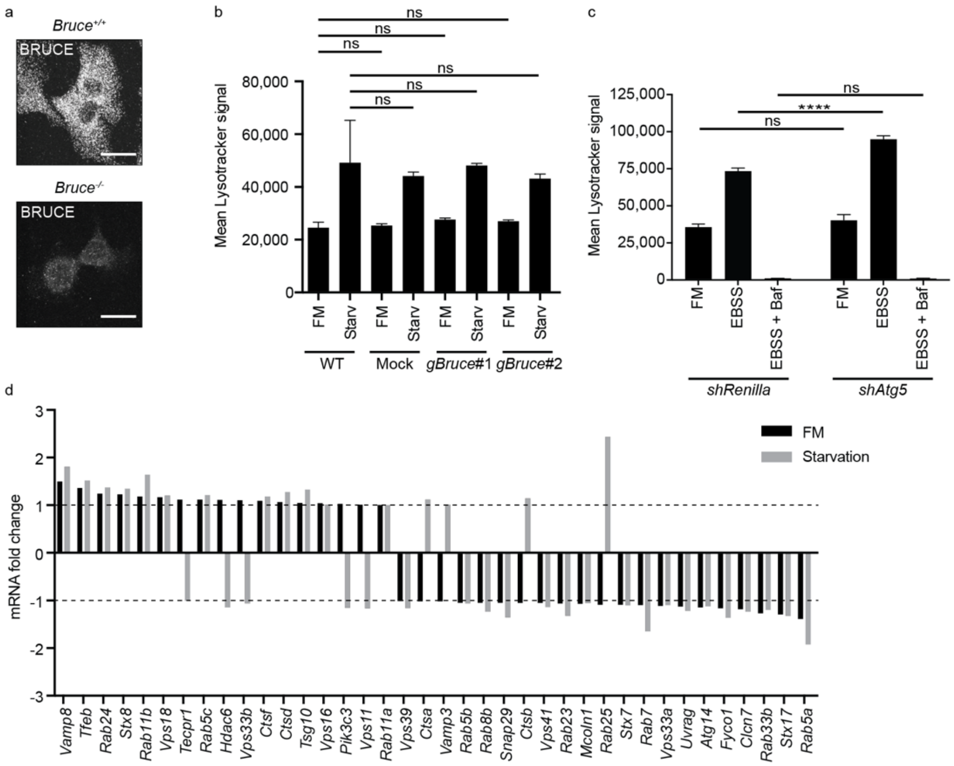

Supplementary Figure 5. Lysosomal pH and lysosomal biogenesis markers are not affected in BRUCE deficient MEFs. (a) Specificity of anti-BRUCE antibody was tested by immunofluorescent staining of *Bruce*$^{+/+}$ and *Bruce* $^{-/-}$ MEFs grown in full medium. **(b)** Mean LysoTracker Red (LTR) signal in BRUCE deficient cells analyzed by flow cytometry. CRISPR/Cas9 BRUCE mutant MEFs (*gBruce#1* and *gBruce#2*) grown in regular and 6 h-starved conditions with or without Bafilomycin A1. Data are presented as mean±SD from three biological replicates (ns: not significant). Representative data are shown from three independent experiments. **(c)** Lysosomal pH in *shAtg5* and *shRenilla* control MEFs was monitored using LTR. Data are presented as mean±SD from three biological replicates (**** p<0.0001, ns, not significant). Representative data are shown from two independent experiments. **(d)** RNA-Seq data of common genes involved in lysosome biogenesis and autophagosome-lysosome fusion. RNA was extracted from *shRenilla* and *shBruce#1* MEFs, grown in FM or starvation medium for 2 h. Fold change of poly (A)-enriched total RNA in *shBruce#1* MEFs was compared to *shRenilla* control MEFs.

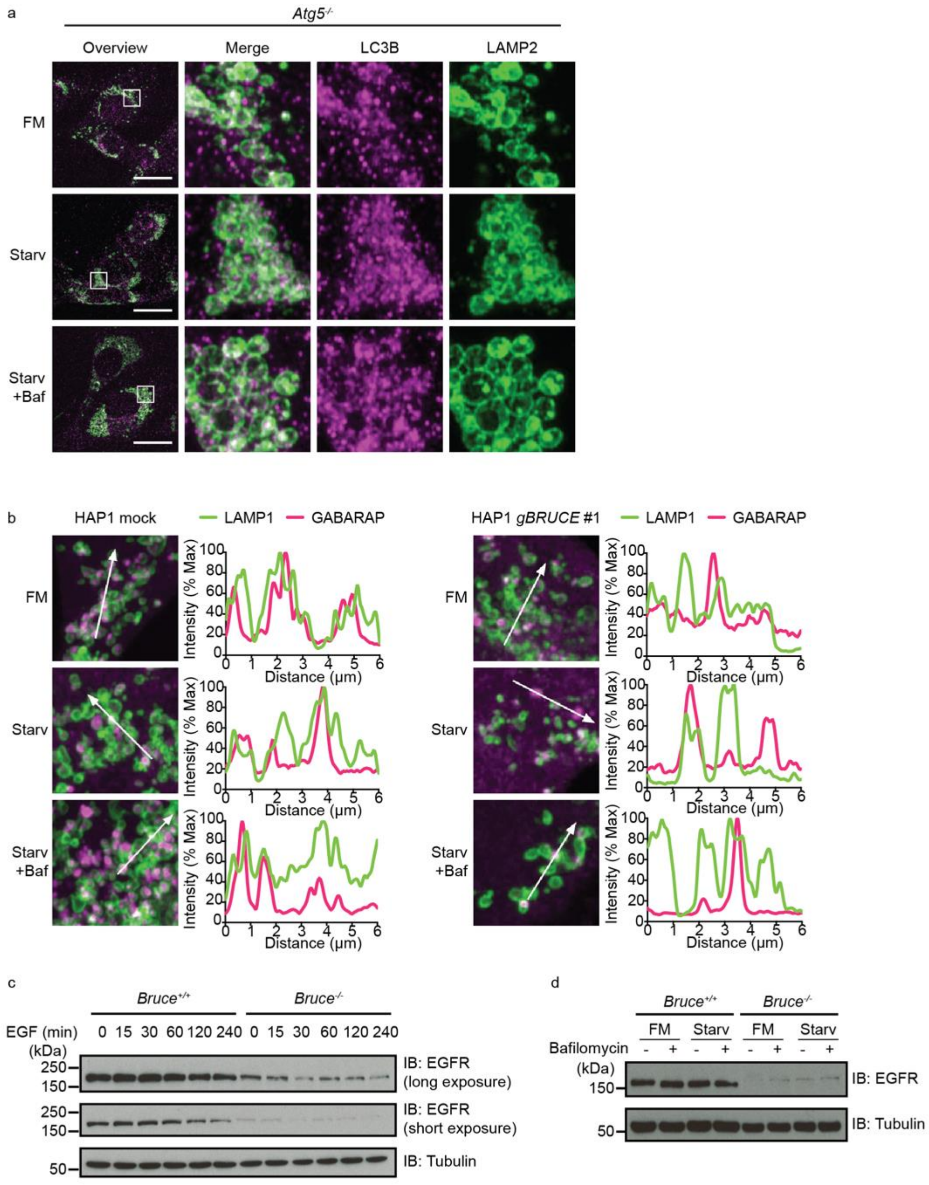

Supplementary Figure 6. Endosomal degradation pathway is not affected in BRUCE-deficient MEFs. (a) Immunofluorescent staining of endogenous LC3B and LAMP2 in *Atg5*$^{-/-}$ MEFs. Cells were grown in fully supplemented medium (FM) or starved for 4 h (Starv) with or without Bafilomycin A1 (Baf; 100 nM). Scale bars, 20 µm. **(b)** Confocal microscopic images of HAP1 cells (mock and g*BRUCE*#1), transiently expressing LAMP1-mGFP and mCherry-GABARAP in FM, or starved for 2 h with or without Bafilomycin A1 (100 nM). Correlating intensity of LAMP1 and GABARAP signals across 6 µm regions marked with arrows is shown in line plots. Scale bars, 20 µm. **(c and d)** Endosomal degradation of epidermal growth factor receptor (EGFR) in *Bruce*$^{+/+}$ and *Bruce*$^{-/-}$ MEFs stimulated with EGF (100 ng/ml) for indicated times (c), in FM or starved for 2 h, with or without Bafilomycin A1 (100 nM) (d). Anti-Tubulin antibody was used for monitoring protein loading.

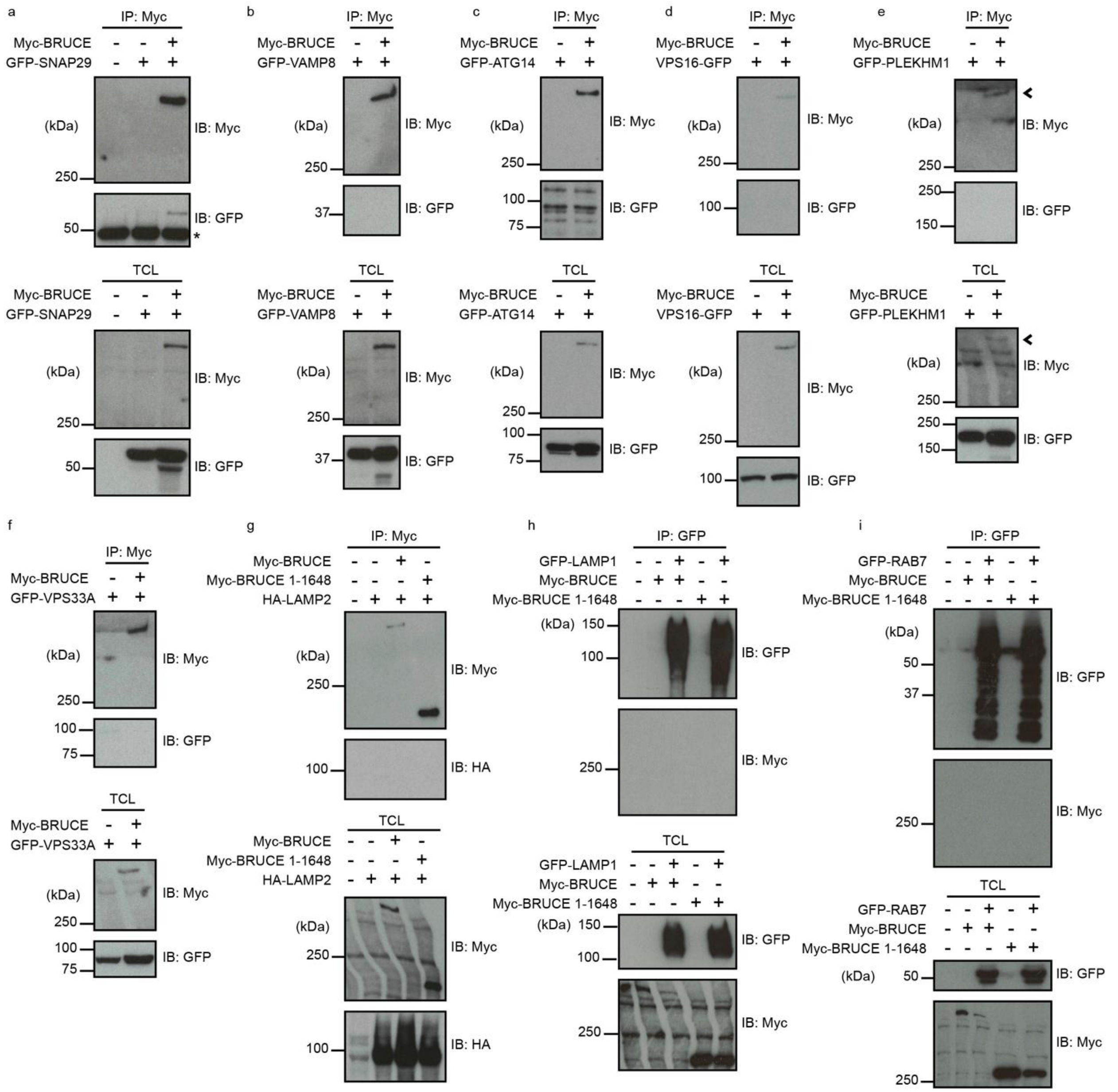

Supplementary Figure 7. Interaction of BRUCE and known regulators of autophagosome-lysosome fusion. (a-i) Co-immunoprecipitation of Myc-BRUCE and known autophagosome-lysosome fusion regulators. Myc-BRUCE WT, 1-1648 and individual regulators were transfected in HEK293T cells. From total cell lysates, Myc-BRUCE was immunoprecipitated using anti-Myc antibody (a-g) and GFP-RAB7 and GFP-LAMP1 were immunoprecipitated using anti-GFP antibody (h and i). Interaction was examined by immunoblotting using the indicated antibodies. *nonspecific band. < indicates band corresponding to Myc-BRUCE WT.

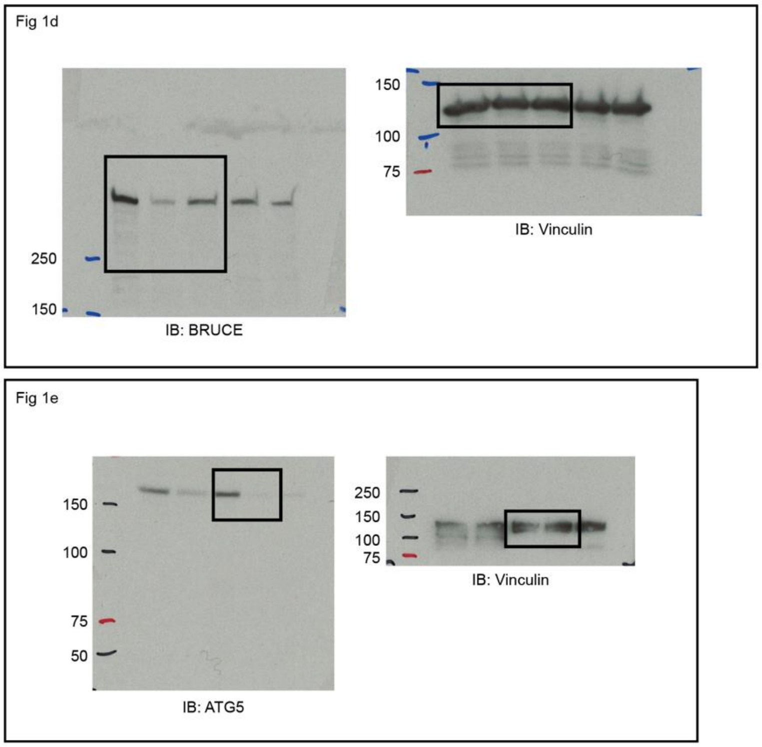

Supplementary Figure 8. Uncropped blots for Figure 1d-e. The regions marked with boxes are presented in the indicated figures.

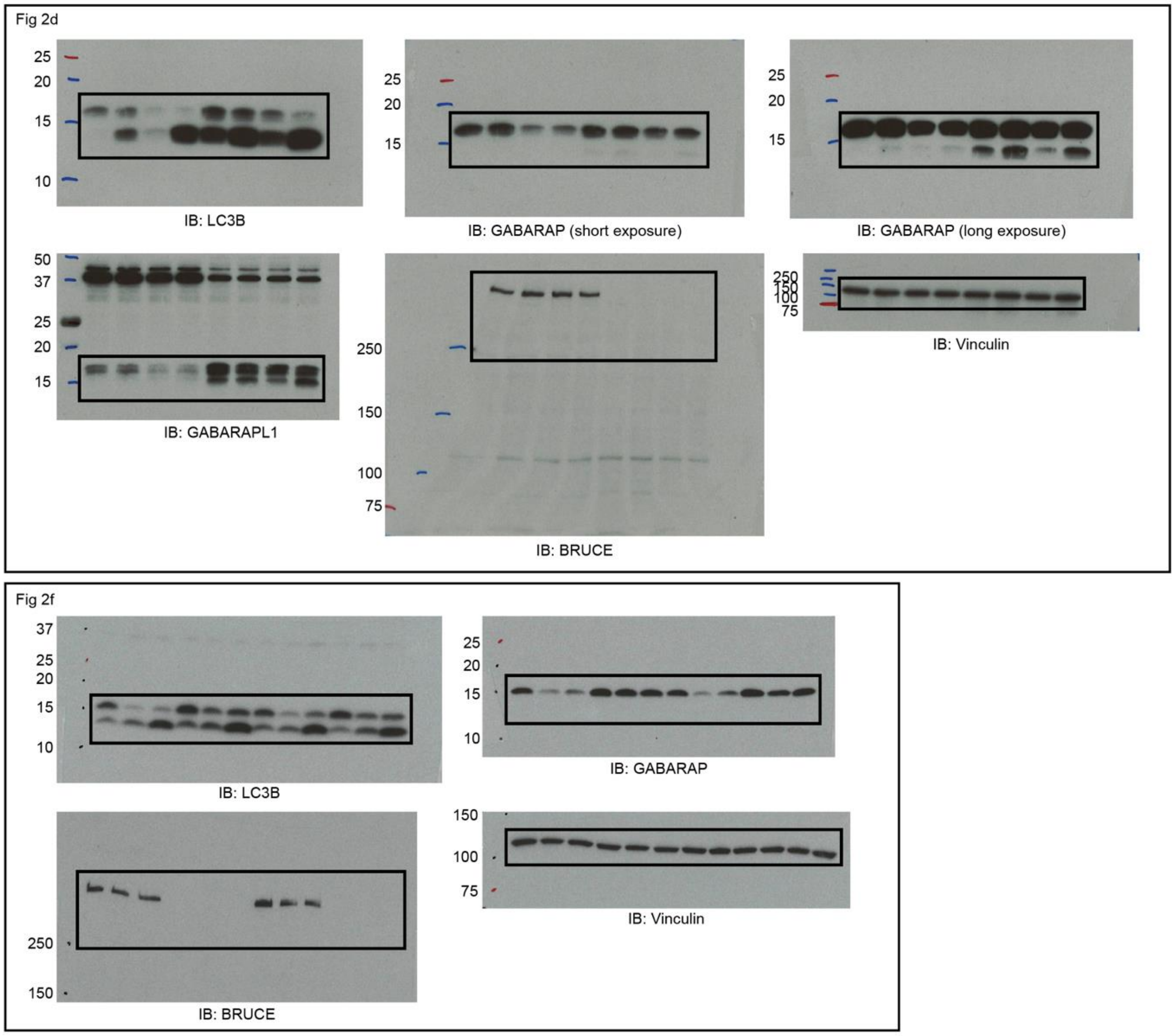

Supplementary Figure 9. Uncropped blots for Figure 2d and 2f. The regions marked with boxes are presented in the indicated figures.

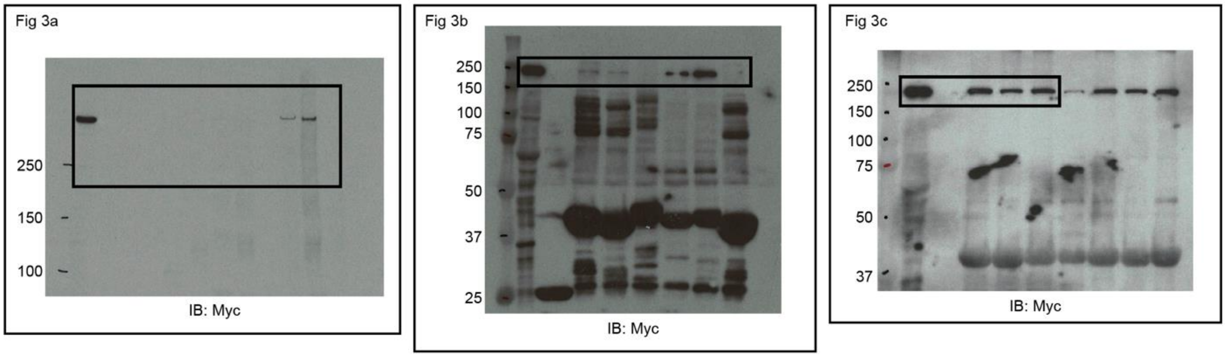

Supplementary Figure 10. Uncropped blots for Figure 3a-c. The regions marked with boxes are presented in the indicated figures.

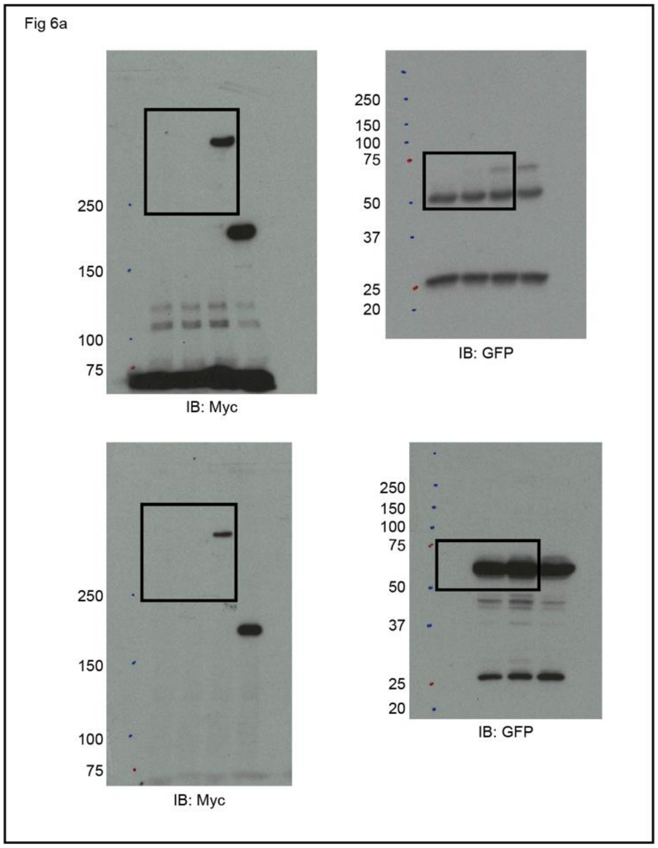

Supplementary Figure 11. Uncropped blots for Figure 6a. The regions marked with boxes are presented in the indicated figures.

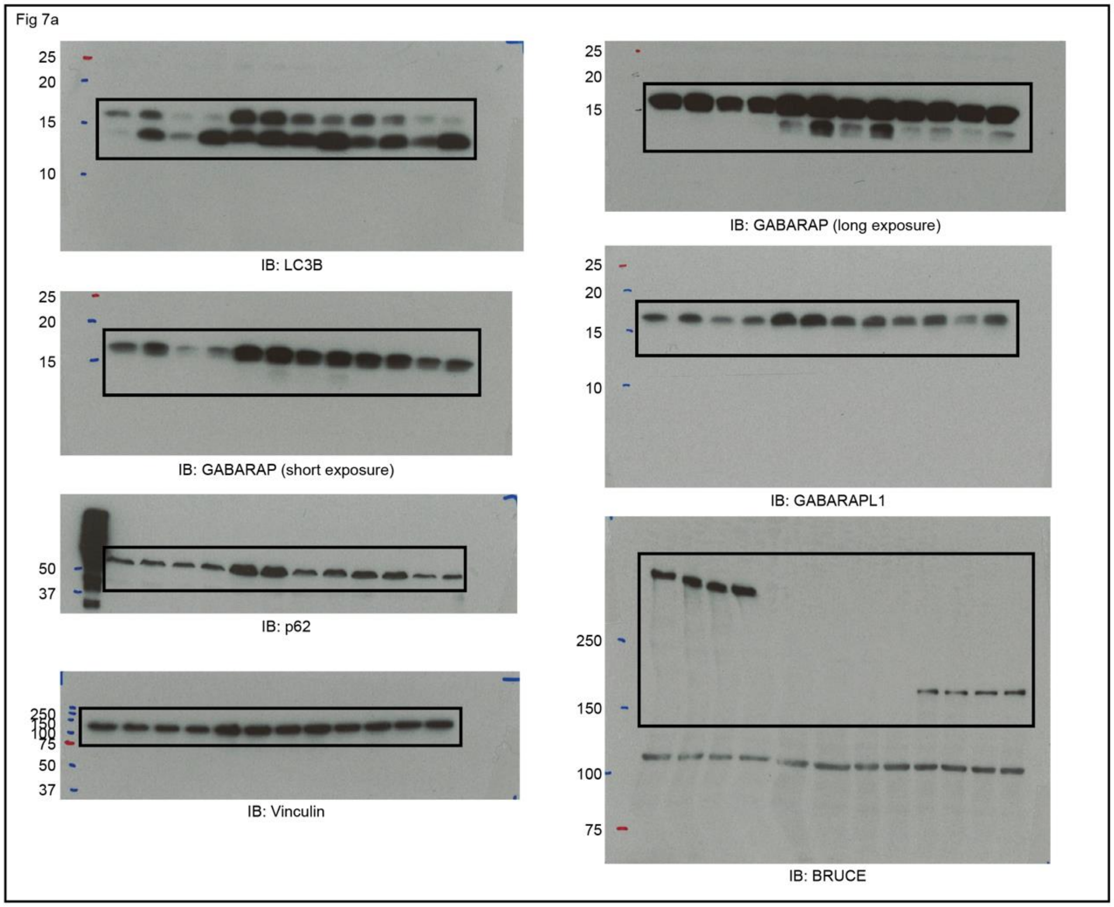

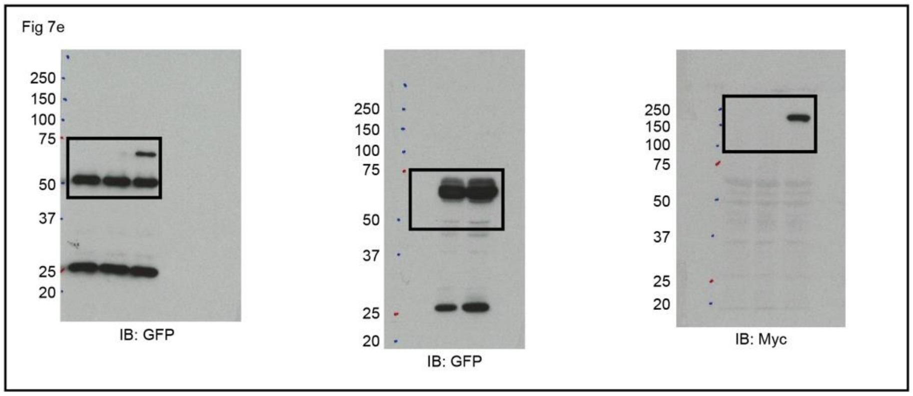

Supplementary Figure 12. Uncropped blots for Figure 7a and 7e. The regions marked with boxes are presented in the indicated figures.
