# Supplementary material for: The IAP family member BRUCE regulates autophagosome–lysosome fusion
Source: Nat Commun. 2018 Feb 9;9:599. doi: 10.1038/s41467-018-02823-x (PMC5807552; doi:10.1038/s41467-018-02823-x)
Supplement: Supplementary file 3 — Description of Additional Supplementary Files [file 41467_2018_2823_MOESM3_ESM.docx]

**File Name:** Supplementary Movie 1

**Description:** STX17-positive mature autophagosomes are formed and expressed in wild type MEFs. WT MEFs stably expressing GFP-STX17 were starved and monitored by live imaging.

**File Name:** Supplementary Movie 2

**Description:** STX17-positive mature autophagosomes are formed and stably expressed in BRUCE knockout MEFs. *Bruce*^-/-^ MEFs stably expressing GFP-STX17 were starved and monitored by live imaging.

**File Name:** Supplementary Data 1

**Description:** Normalized NGS reads and fold change of shRNAs in GFP high vs GFP low gate.
